# Ovary activation does not correlate with pollen and nectar foraging specialization in the bumblebee *Bombus impatiens*

**DOI:** 10.7717/peerj.4415

**Published:** 2018-02-20

**Authors:** Meagan A. Simons, Adam R. Smith

**Affiliations:** Department of Biological Sciences, George Washington University, Washington, D.C., United States of America

**Keywords:** Division of labor, Foraging specialization, Reproductive ground plan hypothesis

## Abstract

Social insect foragers may specialize on certain resource types. Specialization on pollen or nectar among honeybee foragers is hypothesized to result from associations between reproductive physiology and sensory tuning that evolved in ancestral solitary bees (the Reproductive Ground-Plan Hypothesis; RGPH). However, the two non-honeybee species studied showed no association between specialization and ovary activation. Here we investigate the bumblebee *B. impatiens* because it has the most extensively studied pollen/nectar specialization of any bumblebee. We show that ovary size does not differ between pollen specialist, nectar specialist, and generalist foragers, contrary to the predictions of the RGPH. However, we also found mixed support for the second prediction of the RGPH, that sensory sensitivity, measured through proboscis extension response (PER), is greater among pollen foragers. We also found a correlation between foraging activity and ovary size, and foraging activity and relative nectar preference, but no correlation between ovary size and nectar preference. In one colony non-foragers had larger ovaries than foragers, supporting the reproductive conflict and work hypothesis, but in the other colony they did not.

## Introduction

Eusocial insect may specialize on the type of resource they collect (reviewed by  Hölldobler & Wilson, 1990;  Bourke, 1999;  Robinson, 1992;  Beshers & Fewell, 2001). Forager specialization can have adaptive colony-level benefits (e.g.,  Oster & Wilson, 1978;  Bourke, 1999;  Jeanson & Weidenmüller, 2014;  Feinerman & Traniello, 2016). Forager specialization can correspond to multiple distinct morphological worker castes (e.g.,  Wilson, 1980), but specialization can be behavioral as well. For instance, foragers of honeybees, stingless bees, and bumblebees may specialize on collecting nectar or pollen, although this specialization is less pronounced in bumblebees (Free, 1960; Sommeijer et al., 1983; Biesmeijer & Tóth, 1998; O’Donnell, Reichardt & Foster, 2000; Hagbery & Nieh, 2012; Smith, Graystock & Hughes, 2016; Oldroyd & Beekman, 2008; Rueppell, Hunggims & Tingek, 2008; Tan et al., 2015; Russell et al., 2017). The mechanisms behind division of labor in foraging are poorly understood ( Bloch et al., 2009). Here we test predictions from two hypotheses linking ovary development and foraging. The first, the reproductive groundplan hypothesis (RGPH), proposes a mechanism for pollen or nectar specialist foraging. The second, the reproductive conflict and work (RCW) hypothesis, links ovarian development to foraging behavior generally, without reference to specialization.

The RGPH proposes a mechanism for the evolution of foraging specialization in honeybees. The RGPH posits that variation in reproductive physiology underlies foraging specialization (Amdam et al., 2004; Amdam et al., 2006; Page et al., 2006; Page, Rueppell & Amdam, 2012; Page & Amdam, 2007). In the hypothesized reproductive groundplan of ancestral solitary bees, reproductive females forage for pollen as a source of protein, and nectar as a source of carbohydrates for self-maintenance when non-reproductive. In derived eusocial species, these ancestral regulatory mechanisms linking reproduction to foraging have been re-purposed for foraging specialization (Amdam et al., 2004; Amdam et al., 2006; Page et al., 2006; Page, Rueppell & Amdam, 2012; Page & Amdam, 2007). In honeybees, individual pollen foraging specialists have larger ovaries with more ovarioles than nectar foragers (although both groups are typically non-reproductive; Amdam et al., 2004; Amdam et al., 2006; Page & Amdam, 2007; Page, Rueppell & Amdam, 2012). Pollen specialist foragers also have more sensitive sensory tuning, reflecting endocrine links between reproductive physiology and foraging behavior. It takes a lower concentration of sugar (or pollen) touched to the antennae to provoke a pollen specialist to extend her proboscis than a nectar forager (the “Proboscis Extension Response”, PER; Page et al., 2006). However, in the only solitary bee studied to date, there is no association between ovary size and sensory sensitivity (Kapheim & Johnson, 2017). The RGPH thus provides a general mechanistic hypothesis for foraging specialization in bees: reproductive physiology is linked to sensory tuning such that variation in reproductive development underlies forager preference, yet the only data that support this link come from *Apis* honeybees.

Despite the broad comparative nature of the RGPH, most studies of pollen/nectar foraging specialization used *A. mellifera* or other species or strains of *Apis* (“anarchic” *A. mellifera* (Oldroyd & Beekman, 2008), queenless *A. cerana* (Rueppell, Hunggims & Tingek, 2008; Tan et al., 2015), and *A. m. capensis* (Roth et al., 2014). Because bumblebees (*Bombus* spp.) also have pollen and nectar specialist foragers, they can be used for a comparative test of RGPH (O’Donnell, Reichardt & Foster, 2000; Hagbery & Nieh, 2012; Smith, Graystock & Hughes, 2016). Forager specialization, defined as individuals that forage on pollen or nectar more frequently than expected relative to the colony as a whole, has been shown in *B. bifarius* (O’Donnell, Reichardt & Foster, 2000), *B. impatiens* (Hagbery & Nieh, 2012; Russell et al., 2017) and *B. terrestris* (Smith, Graystock & Hughes, 2016). Hagbery & Nieh (2012) showed that preferences in *B. impatiens* were lifelong, but Russell et al. (2017) reported short-term specialization. Specialization is a continuum: most bees forage for some pollen, all bees at least occasionally forage for nectar, and many are generalists (Verhaeghe et al., 1999; O’Donnell, Reichardt & Foster, 2000; Hagbery & Nieh, 2012; Konzmann & Lunau, 2014; Smith, Graystock & Hughes, 2016; Russell et al., 2017). Smith, Graystock & Hughes (2016) explicitly tested whether ovary activation was linked to foraging specialization in *B. terrestis.* While they did not find the striking links between ovary development and foraging specialization seen in honeybees, some of their colonies did show correlations between ovary activation and pollen foraging or sensory sensitivity (Smith, Graystock & Hughes, 2016). In this study, we use the same methods as Smith, Graystock & Hughes (2016) to test whether pollen and nectar foraging specialization in *B. impatiens* is linked to ovary activation. We use *B. impatiens* because it is the species in which foraging specialization has been most thoroughly demonstrated (Hagbery & Nieh, 2012) and because the mixed results of Smith, Graystock & Hughes (2016) suggest that further study is warranted to test ovary-foraging links.

In this study we used two lab colonies given access to *ad lib* sugar water (artificial nectar) and pollen in separate feeders to quantify foraging specialization. After foraging observations, we tested the PER of bees at four different sugar concentrations. Lastly, we collected bees for dissection and ovary size measurement. We test the prediction that pollen specialist foragers of the bumblebee *B. impatiens* have larger ovaries than nectar specialists. We also test the hypothesis that both ovary activation and pollen specialist behavior will correlate with increased sensitivity, as measured through PER. PER is less informative of innate sensory sensitivity in bumblebees than in honeybees because bumblebees do not extend their proboscis to low concentrations of sucrose without training ( Laloi et al., 1999;  Riveros & Gronenberg, 2009). Nevertheless, because Smith, Graystock & Hughes (2016) found that in one of their colonies PER measures of sensitivity correlated with ovary activation, we also include PER tests in this study.

Another hypothesis linking ovary activation to foraging, which is unrelated and non-exclusive to the RGPH, is the ‘Reproductive Conflict and Work’ hypothesis. This hypothesis proposes that potentially reproductive workers avoid foraging in order to save their energy for reproduction (Schmid-Hempel, 1990; Roth et al., 2014). In the cape honeybee (*A. mellifera capensis*) (Roth et al., 2014), *B. impatiens* (Jandt & Dornhaus, 2011), and *B. terrestris* (Smith, Graystock & Hughes, 2016) ovary activation correlated with reduced foraging rate. This hypothesis predicts that non-foragers will have larger ovaries than foragers, whatever their specialization, and is unrelated to the RGPH prediction that among active foragers, pollen specialists will have more developed ovaries than nectar specialists.

## Methods

Two bumblebee colonies of unknown age were obtained from Koppert Biological Systems, and were queenright and without males or gynes; the queen was present during the entire study. Colony 1 contained 113 workers at collection, and colony 2 contained 132 workers. We began observations on each colony within two weeks of receipt from Koppert. For each colony, the nest box containing the hive as shipped from Koppert was placed within a plastic flight cage (79.5 cm × 39.5 cm × 25 cm; length × width × height measure our boxes). The colony was placed against one end of the flight cage, petri dishes with 1.5 M sucrose (nectar feeder) or pollen were placed against the opposite end, and one feeder was in each corner following Hagbery & Nieh (2012). We used pollen supplied by Koppert that had originally been collected by honeybees. All pollen was from the same batch, and was briefly ground in an electric coffee grinder to break up the clumps formed by the honeybees when they packed the pollen onto their corbicula before the pollen was taken from them. We marked all bees except the queens with numbered colored disks glued on their thorax with Krazy Glue gel, a polyacrylamide adhesive. Bees were removed from the colony with foreceps and refrigerated to enable marking. Colonies were kept at room temperature (∼22 C).

We allowed each colony to forage for one hour per day, following Hagbery & Nieh (2012), during which time we videotaped all feeding events at both feeders; one observation period of colony 1 lasted for 1.5 h. Foraging observations were begun at 1 pm each day. During other times the bees were in their dark nest box without food. We recorded the identity of all individuals seen actively gathering pollen (defined as pulling pollen toward their body with their legs, walking across the pollen while dragging their abdomen in it (Russell & Papaj, 2016) and/or grooming pollen onto their corbicula) or drinking nectar (proboscis extended into the sugar water). We recorded all individuals foraging during scans at one-minute intervals. Individuals that left and returned during a scan were only counted once, and an individual could be counted in consecutive scans without returning to the nest if they were still (or again) gathering pollen or imbibing nectar. We observed colony 1 for 5.5 h between 21 and 27 October 2014, and colony 2 for 5 h between 26 February and 3 March 2015. Hagbery & Nieh (2012) showed that foraging behavior over a few days correlates with lifelong specialization. We collected bees for PER directly from the nest box after foraging observations ended. We measured thorax width (intertegular span) using electronic calipers as a measure of body size (Cane, 1987).

### PER

We followed exactly the methods of Smith, Graystock & Hughes (2016), briefly: bees were placed in PER harnesses, fed for 30 min until satiation on 1.5 M sucrose, and then starved for 4 h following Graystock et al. (2013),  Graystock et al. (2016) and Smith, Graystock & Hughes (2016). Our test were done under dim red light to avoid visual stimulation, and used sucrose concentrations that were substantially higher than those used in previous studies of honeybees because few bumblebees respond to low concentrations of sucrose without repeated trials ( Laloi et al., 1999;  Riveros & Gronenberg, 2009; Smith, Graystock & Hughes, 2016). We presented bees with sucrose solutions of 60, 70, 80, and 90% w/v, presented in increasing concentration with 20 min between trials. A positive response consisted of an extended proboscis when a ball of cotton wool soaked in sucrose solution was touched to the bee’s antennae. Each bee received a PER score that was the sum of its positive responses (0–4).

### Ovary measurement

Bees were placed into 70% ethanol, and dissections took place in 70% ethanol at 10× magnification. The width of the largest oocyte of either ovary was measured using an ocular micrometer following standard methods (e.g., Martins & Serrão, 2004; Biani & Wcislo, 2007; Cini, Meconcelli & Cervo, 2013). Ovariole number, which is fixed during adult development, varies among honeybee individuals (Amdam et al., 2004; Amdam et al., 2006; Page & Amdam, 2007; Page, Rueppell & Amdam, 2012; but see Ronai et al., 2017). However all non-parasitic *Bombus* have eight ovarioles ( Cumber, 1949;  Iwata, 1955;  Amsalem et al., 2015), so here we examine ovary activation (enlarged oocytes or not), and use the width of the largest oocyte as our measure of activation for each bee. In the honeybee literature ovary ‘size’ is often used as a synonym for ovariole number (e.g., Ronai et al., 2017). Here we use it to refer to the actual size of the ovaries, which varies greatly between non-reproductive and reproductively active individuals (e.g., Michener, 1974). Ovary size in *B. impatiens* can change during adult life (e.g., Jandt & Dornhaus, 2011). Because ovary size and body size were correlated (see ‘Results’), we also use the variable “ovary size relative to body size”, calculated by dividing oocyte width by thorax width.

### Statistical analyses

Foraging specialists were determined by using a binomial test following O’Donnell, Reichardt & Foster (2000). Those bees that significantly (exact binomial test two-tailed *p* < 0.05) deviated from expected values (computed from colony total number of nectar and pollen observations) were categorized as specialists; other foragers were categorized as generalists. The proportion of nectar to pollen observations differed between colonies, so the expected values used to calculate specialization did as well. Only bees with at least 10 total foraging observations were included in the specialization categories. Statistics were computed in SPSS 23, except for the exact binomial tests, which were computed in R. Differences between forager categories (pollen specialists, nectar specialists, generalists, non-foragers, and bees with <10 foraging observations) in ovary size, were analyzed using a generalized linear model (GZLM) with a gamma distribution and a log link function (to account for the positive skew in ovary size distribution), with colony and foraging category factors, and body size as a covariate. We also measured foraging specialization as a continuous measure: percent of observed scans on the nectar feeder. As above, only bees with at least 10 total foraging observations were included. Bivariate correlations were calculated using Spearman’s rank correlations. We used a similar GZLM to analyze PER scores, with colony and foraging category factors, and body size and ovary size as covariates.

## Results

### Foraging specialization

**Figure 1:**
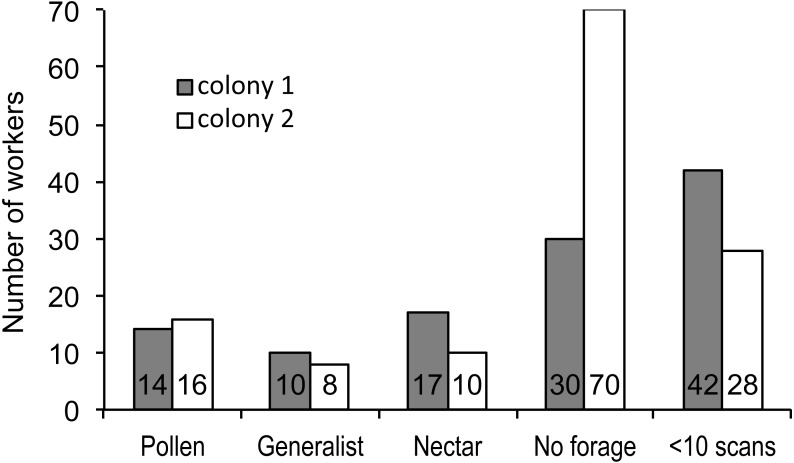
Foraging specialization by colony. Distribution of bees in each colony among specialist or generalist categories (for bees with at least 10 scan observations), bees that never foraged, and bees that foraged, but were recorded in fewer than 10 scans. The number of bees in each category is listed at the base of each bar.

**Figure 2:**
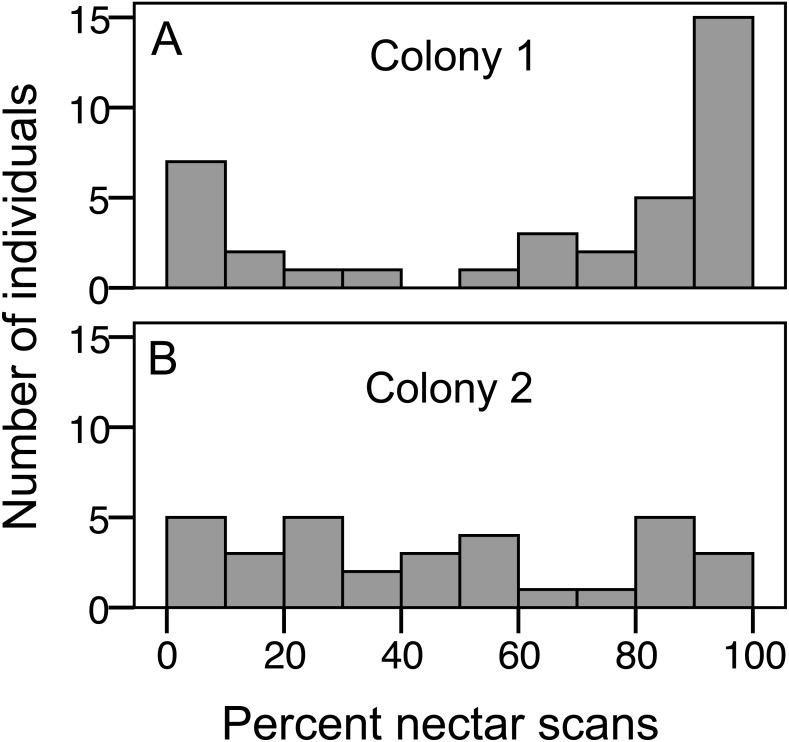
Variation in nectar preference among foragers. (A) Colony 1; (B) Colony 2. The number of individuals (*y* axis) in each colony with a given total percentage of nectar observations. Only individuals with at least 10 foraging observations are included.

We recorded 3,618 visits at our feeders. Among the foragers (at least 10 recorded foraging scans) the mean = 45.4 ± 33.0 SD, median = 39, range = 10–142. Both colonies contained both nectar and pollen specialists, as well as individuals that never foraged (Fig. 1). Foragers with fewer than 10 observations were not assigned to a foraging or non-foraging category (Fig. 1). In both colonies, specialists were more common than generalists, but the distribution of bees was different between colonies (Fig. 1; ${\chi }_{\mathrm{1,4}}^{2}=19.61$, *P* < 0.001). The colony differences are due to the difference in non-foraging bees. When only the three foraging categories are considered, there was no difference (${\chi }_{\mathrm{1,2}}^{2}=1.53$, *P* = 0.47). Despite the similar distribution of foraging generalists and specialists in both colonies, foraging preference was not similarly distributed. Colony 1 showed a bimodal distribution of preference (measured as percent of all scans at the nectar feeder for each bee), while colony 2 showed an even distribution (Fig. 2). Colony 1 showed a stronger preference for nectar than did colony 2 (79.5% of 2,230 scans vs. 55.5% of 1,388 scans; ${\chi }_{\mathrm{1,1}}^{2}=237.58$, *P* < 0.001). Among those bees with at least 10 scans, the median percent nectar ± interquartile range was 84.1 ± 62.6, and 47.4 ± 64.7 for colonies 1 and 2, respectively. Because the binomial tests for specialization were calculated relative to the colony means, generalist foragers in colony 1 had a significantly stronger preference for pollen than those in colony 2 (Mann Whitney *U* = 2.0, *n* = 18, *P* < 0.001; pollen and nectar specialists did not differ between colonies).

**Figure 3:**
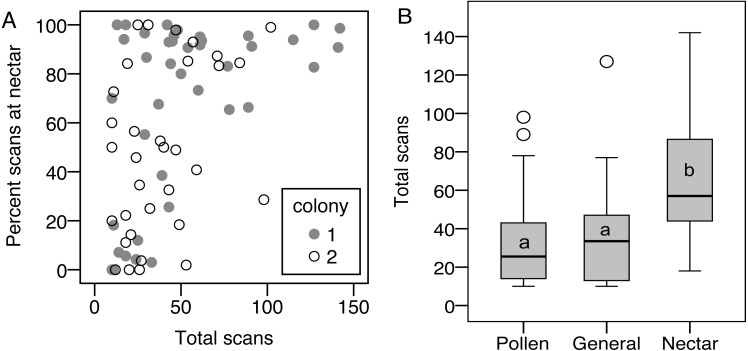
Nectar foragers are more active than other foragers. (A) Foraging activity, measured as number of scans observed on a feeder, correlates with nectar preference, measured as the percentage of each bee’s foraging observations on the nectar feeder. (B) As a result, nectar foragers were more active than the other two foraging groups. Bars with different letters are significantly different.

Limiting the analysis to only the foragers (nectar specialists, pollen specialists, and generalists) allows analysis of specialization, i.e., percentage of scans at the nectar feeder, as a continuous variable. There was a correlation between total scans and percent nectar, showing that the most active foragers tended to be nectar specialists, while less active foragers showed the full range of nectar preference (*σ* = 0.47, *N* = 75, *p* < 0.001, correlations also significant when analyzed for each colony separately; Fig. 3). As a result, nectar foragers were significantly more active than the other two groups (LSD pairwise *P* < 0.001 and *P* = 0.002 for pollen specialists and generalists, respectively; Fig. 3). Body size did not correlate with total scans or percent nectar (*N* = 58, *σ* = 0.06, *p* = 0.67 and *σ* =  − 0.06, *p* = 0.68, respectively).

### Ovary size

**Figure 4:**
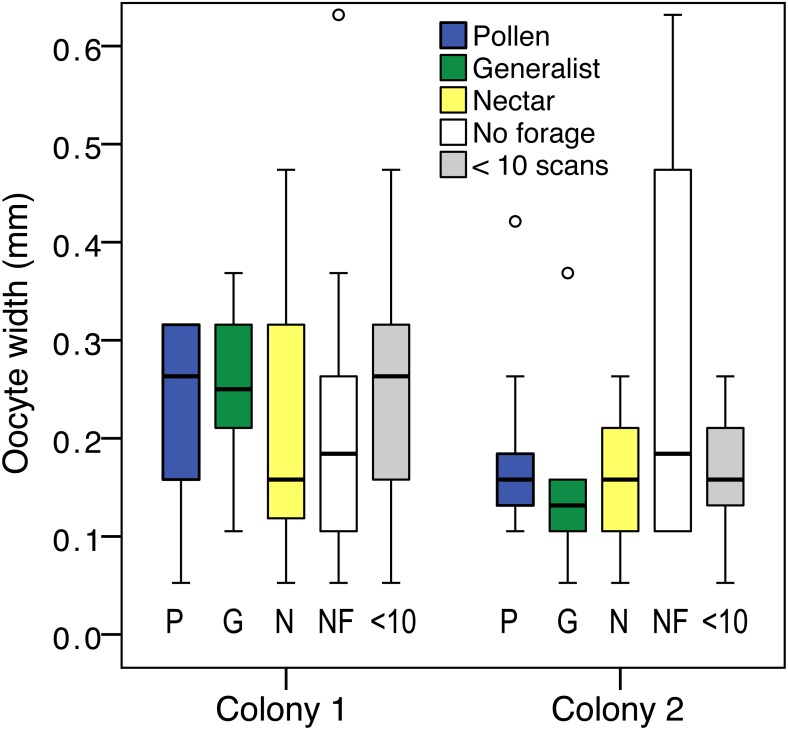
Ovary size variation. Boxplot of ovary size for: pollen specialists (P), generalists (G), nectar specialists (N), non-foragers (NF) and bees with fewer than 10 scans (<10) by colony. There is a significant difference between colonies, and non-foragers have significantly larger ovaries than all other colony 2 foraging groups.

Ovary size differed between foraging categories (pollen specialist, generalist, nectar specialist, and bees with <10 scans; LR ${\chi }_{4}^{2}=10.97$, *P* = 0.03). There was also an effect of colony (LR ${\chi }_{1}^{2}=17.85$, *P* < 0.001; Fig. 4), and an effect of body size (LR ${\chi }_{1}^{2}=27.61$, *P* < 0.001). There was a significant colony by group interaction, driven by the large ovaries of colony 2 non-foragers (LR ${\chi }_{4}^{2}=10.49$, *P* = 0.03). Non-foragers had significantly larger ovaries than pollen foragers (pairwise LSD test *P* = 0.03) and nectar foragers (*P* = 0.01), nearly significantly larger ovaries than generalist foragers (*P* = 0.05). There were no other significant pairwise differences between foraging categories. The differences between foraging categories were a result of the large ovaries of colony 2 non-foragers, as indicated by the significant interaction effect. There was no significant effect of group when colony 1 was analyzed alone (LR ${\chi }_{4}^{2}=2.06$, *P* = 0.72).

Body size correlated with ovary size (*N* = 119, *σ* = 0.33, *p* < 0.001), but did not differ between foraging categories (oneway ANOVA *F*_4,119_ = 0.15, *P* = 0.96). Ovary size measured relative to body size did not differ by foraging group (LR ${\chi }_{4}^{2}=6.96$, *P* = 0.14), but the effect of colony (LR ${\chi }_{4}^{2}=10.55$, *P* = 0.001) and colony by group interaction (LR ${\chi }_{4}^{2}=13.07$, *P* = 0.01) remained.

Analysis of percent observations at the nectar feeder for foragers only showed no correlations between percent nectar and ovary size or ovary size relative to body size. There was a correlation between foraging activity, measured as the total number of scans, and ovary size (*σ* = 0.36, *N* = 59, *p* = 0.005). Results were similar for ovary size relative to body size (*σ* = 0.37, *N* = 59, *p* = 0.005). Foraging activity did not differ between colonies (*t*_174_ = 7.00, *P* = 0.84).

### PER

**Figure 5:**
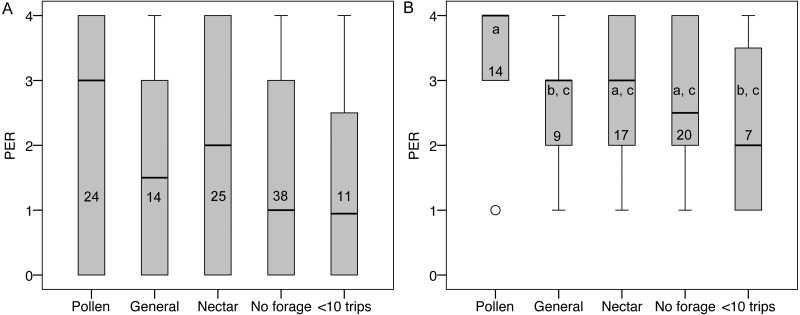
PER scores and forager categories. (A) Boxplots of PER scores for each foraging category. There were no significant differences between groups. (B) Boxplots of PER scores with zeros excluded. Bars that share a letter are not significantly different. Numbers on each bar indicate sample size for each group.

PER score did not differ between groups (Fig. 5), nor was there an effect of colony, body size, ovary size, or ovary size relative to body size. However, many bees had a PER score of zero (45 of 112), suggesting that they were not responsive to the test. Zero scores were not concentrated in any foraging group relative to non-zero scores (${\chi }_{4}^{2}=1.72$, *P* = 0.79). When scores of zero were excluded, there were still no significant overall effects, but pollen foragers had higher sensory sensitivity than generalist foragers (LSD pairwise *P* = 0.048; Fig. 5). There were no significant correlations between PER score and ovary size or ovary size relative to body size, either with all bees or only non-zero PER bees.

## Discussion

Here we show that there is no association between ovary activation and foraging specialization in *B. impatiens,* similar to our previous work in *B. terrestris* (Smith, Graystock & Hughes, 2016). This is contrary to the predictions of the RGPH. We did find an association between ovary activation and foraging activity, as well as an association between foraging activity and nectar specialization, but there was no association between specialization and ovary size, and no indication that pollen, rather than nectar, foragers had more developed ovaries, as they do in *A. mellifera* (Amdam et al., 2004; Amdam et al., 2006; Page et al., 2006; Page, Rueppell & Amdam, 2012; Page & Amdam, 2007). Rueppell, Hunggims & Tingek (2008) found that pollen foragers had larger ovaries than nectar foragers in queenless *A. cerana* nests, but a subsequent study on the same species found no difference between the two forager types (Tan et al., 2015). Ovary size has not been measured in the context of pollen and nectar foraging specialization in any other species.

We found a sensory sensitivity difference consistent with *A. mellifera.* Our initial PER results show no difference in sensory sensitivity between groups, however, when the data are analyzed only using bees that responded to the PER stimulus, then pollen foragers showed higher sensory sensitivity, consistent with previous work in *A. mellifera* (Page et al., 2006; Page, Rueppell & Amdam, 2012; Page & Amdam, 2007). We believe that it is reasonable to exclude the zeros from the PER analysis because the sucrose concentrations we used (60–90%) are so high that all bees should be expected to respond. However, this highlights a fundamental problem for using PER to test sensory sensitivity in bumblebees: they only show the untrained PER response to high concentrations of sucrose ( Laloi et al., 1999;  Riveros & Gronenberg, 2009). We used PER here to be consistent with previous studies of *A. melifera*, but the assay simply does not have the power to detect sensory sensitivity differences in bumblebees that it does in honeybees, where subtle differences in stimuli can be assayed (e.g., Page et al., 2006). Our results suggest that there may be differences in sensory sensitivity between pollen specialists and generalist foragers, but other PER methods may better test this hypothesis (e.g., Ma et al., 2016; Muth et al., 2017; Kapheim & Johnson, 2017). Perhaps more direct measures, such as electrophysiological recordings of antennal stimulation, or foraging choice experiments at a range of feeders, could provide a more powerful measure of sensory sensitivity variation in bumblebees.

Regardless of the PER results, we show that there was substantial variation in ovary activation and foraging specialization, but that there was no association between these two variables, consistent with our previous work on *B. terrestris* (Smith, Graystock & Hughes, 2016). Further research into the mechanisms of specialization in bumblebees, as well as comparative studies with stingless bees, would provide greater insight into the evolution of specialist foraging. For instance, foraging behavior in bumblebees is strongly dependent on individual learning (Goulson, 2010), and intra-individual variation in behavior is common in social insects (reviewed by  Jandt et al., 2014;  Jeanson & Weidenmüller, 2014). Individual specialization may be influenced by early foraging rewards, perhaps interacting with existing individual variation, shaping later behavior. Additionally, our results also suggest interactions of individual and colony level influences on foraging behavior. In our study, colony 1 foraged for relatively more nectar than colony 2, which resulted in more nectar-biased generalist foragers. Nevertheless, there were still pollen specialists in colony 1, and nectar specialists in colony 2. A limitation of our study is that we used only two colonies, both of unknown age. Previous studies of specialization in bumblebees also show colony-level variation in nectar preference, the percent of foragers that are specialists, PER sensitivity, and average ovary size. Future studies that explicitly test for interactions between individual variation, colony foraging needs, and colony age would be productive (O’Donnell, Reichardt & Foster, 2000; Hagbery & Nieh, 2012; Smith, Graystock & Hughes, 2016; Russell et al., 2017).

Our data offer mixed support for the “Reproductive Conflict and Work” hypothesis, which predicts that workers with more developed ovaries avoid foraging effort (Schmid-Hempel, 1990; Roth et al., 2014). Non-foragers in colony 2 had larger ovaries than foragers, but this relationship was not seen in colony 1. Smith, Graystock & Hughes (2016) showed that non-foraging *B. terrestris* had larger ovaries than foragers, and in *B. impatiens* non-foraging workers were more likely than foragers to enlarge their ovaries following queen removal (Jandt & Dornhaus, 2011), or have larger ovaries when the colony naturally entered the competition phase (Foster et al., 2004). However, among foragers, we found a positive correlation between ovary size and foraging activity, suggesting that while some individuals may avoid foraging effort to invest in ovary development, there is not a direct inverse relationship between the two. Moreover, the effort involved in foraging in our flight cages was minimal, and involved no exposure to natural predators or pathogens.

## Conclusions

In the three species studied in detail, bumblebees exhibit pollen and nectar specialization among foragers (O’Donnell, Reichardt & Foster, 2000; Hagbery & Nieh, 2012; Smith, Graystock & Hughes, 2016; Russell et al., 2017, this study). Yet this study and Smith, Graystock & Hughes (2016) find no evidence for an association between ovary development and foraging preference, as predicted by the RGPH. Russell et al. (2017) found no differences in antennal sensory morphology, and Kapheim & Johnson (2017) found differences in the Dufour’s gland, but not ovaries, of the solitary bee *Nomia melanderi.* This suggests that bumblebees may have evolved a similar phenotype—pollen and nectar specialist foragers—using different mechanisms than honeybees. We propose that future work focused on individual experience and learning, colony resource needs, and colony developmental stage, rather than ovary activation, will provide novel insights into the expression of pollen and nectar specialization in bumblebees.

## Supplemental Information

10.7717/peerj.4415/supp-1 Data S1 Raw data

## Additional Information and Declarations

### Data Availability

The following information was supplied regarding data availability: The raw data are uploaded in Data S1.
